# Relativistic independence and multipartite quantum correlations

**DOI:** 10.1140/epjs/s11734-025-01836-0

**Published:** 2025-08-11

**Authors:** Rain Lenny, Amit Te’eni, Michael Suleymanov, Eliahu Cohen

**Affiliations:** Faculty of Engineering and Institute of Nanotechnology and Advanced Materials, Bar-Ilan University, Max VeAnna Webb, Ramat Gan, 5290002 Israel

## Abstract

Multipartite quantum correlations are complex and intricate, posing challenges for their detection and quantification. Nevertheless, they are central to both fundamental quantum science and its numerous applications. We discuss several approaches for analyzing quantum correlations, with an emphasis on the Relativistic Independence framework which allows one to detect and differentiate classes of multipartite entangled states, while also yielding refined quantum bounds on multipartite systems. This framework is shown to rely on a recently demonstrated tradeoff between local and nonlocal correlations, signifying the enabling power of quantum uncertainty. Furthermore, we examine these notions from the broader perspective of quantum reference frames (QRFs), attempting to see which of the above concepts are perspective-dependent and which are not. Large parts of the current discussion, as well as many of the quantitative results presented, specifically rely on the structure of the covariance matrix, whose determinant remains invariant under QRF changes, thereby signaling its fundamental significance.

## Introduction

The correlations that can arise in quantum theory are much more intricate and interesting than those that can arise in classical physics. Thanks to quantum entanglement, measurements made on distant systems can lead to nonlocal correlations—correlations that are classically inexplicable, i.e. are incompatible with local-realism. To be concrete, consider a system shared by *N* parties. The *n*th party has the subsystem $A^{(n)}$, with the corresponding Hilbert space $\mathcal {H}_n$. The Hilbert space of the total system has a fixed tensor product structure:

1 $$\begin{aligned} \mathcal {H} = \bigotimes _{n=1}^N \mathcal {H}_n . \end{aligned}$$

For $N=2$, we say the system is *bipartite*. For $N>2$, it is *multipartite*. Multipartite systems are subtler than their bipartite counterparts. While a bipartite state is either separable or entangled, the multipartite scenario exhibits further types of nonlocality.

Entanglement importantly entails many fundamental [1, 2] and applicative consequences. It allows one to perform teleportation [3], nonlocal measurements [4–6], superdense coding [7], secure communication [8], quantum-enhanced metrology [9] and numerous other tasks. However, the role of quantum entanglement in quantum computation, and in particular, its contribution to the quantum computational speedup are less clear [10–13]. Also puzzling is its role in quantum illumination [14, 15] and quantum radar [16]. Multipartite entanglement is used for quantum secret sharing [17]; key distribution [18]; one-way [19] and distributed [20] quantum computing; multiplayer quantum games [21]; quantum communication [22] and metrology [23]. It is therefore important to properly detect and characterize entangled states [24].

Much effort has been devoted over the last few decades to characterize the set of quantum correlations [25–27]. On the one hand, a complete characterization of quantum correlations has been found, given by the infinite, yet convergent Navascués- Pironio-Acín (NPA) hierarchy of semi-definite programs [28], which must be satisfied by all quantum correlations. On the other hand, many researchers have been trying to understand the structure of quantum correlations. To do so, various (mostly information-theoretic) principles have been suggested for ruling out stronger-than-quantum correlations. Notable examples include the non-trivialization of communication complexity [29, 30], Macroscopic Locality [31], Information Causality [32], the Uncertainty-Steering correspondence [33] and Local Orthogonality [34].

Another principle was suggested in [35] which was termed Relativistic Independence (RI). This principle is a conjunction of two fundamental requirements we would like any theory of nature to satisfy – relativistic causality (which implies a finite speed for any type of information transfer) and generalized uncertainty. The latter is an extension of quantum uncertainty to multipartite systems, applicable to any statistically-meaningful theory. Based solely on this construction we have derived several known bounds on quantum correlations, including Tsirelson’s bound [36] (thereby ruling out Popescu-Rohrlich (PR) boxes [37] and other stronger-than-quantum models) and the TLM bound [38–40], as well as other novel bounds, including richer, more detailed versions of the above bounds. Our approach has already proved useful for deriving new multiplicative Bell inequalities and their Tsirelson bounds [41], as well as new bounds on correlations within signaling, non-Hermitian systems [42]. Our approach can be easily generalized, and indeed within [43, 44] and on-going works we have been analyzing bounds on multipartite entanglement in multiple-input-multiple-output scenarios. Furthermore, in [45] we applied this approach to correlations in time, thereby suggesting new bounds of the Leggett-Garg form [46]. RI was also shown to enable entanglement detection in bipartite [47] and multipartite systems [48].

Another aspect we would like to address in this review is the perspective-dependence of quantum correlations in the *relational* formulation of quantum mechanics, particularly in the *quantum reference frames* (QRFs) approach. Frames of reference play a central role in physical theories, since the covariance of the laws of nature is expressed in terms of transformations between reference frames. The relational approach suggests treating reference frames as physical objects obeying the same laws as the described objects. The assumption that nature is fundamentally quantum naturally leads to the QRF formalism, introduced in [49–51] and further developed in [52–87]. Several different approaches have been developed throughout the years, namely the *operational* [66–69], *informational* [58–65], *perspectival* [70–73] and *perspective-neutral* [74–81].

In this review article, we discuss bipartite and multipartite correlations from various points of view. We present inequalities that delineate local-hidden-variable models as well as quantum models. The former type of inequalities may be used to detect entanglement—an important task, given the utility of entanglement for the many aforementioned applications. On the other hand, quantum bounds have a deep fundamental significance, as they illuminate the limitations of quantum resources. We demonstrate how these inequalities are refined via the RI framework. We also discuss multipartite correlations within the context of relational quantum mechanics.

The manuscript is structured as follows. Section 2 introduces Bell inequalities and quantum bounds for bipartite systems. Section 3 introduces the concept of RI and its implications. Section 4 demonstrates the refinement of quantum bounds via RI. It also introduces a new kind of Bell inequalities, which were derived directly from the RI framework. Section 5 reviews bounds for multipartite systems. Again, we also refine these bounds using our framework. Section 6 discusses multipartite correlations and their bounds from the perspective of quantum reference frames. Section 7 summarizes this review and outlines directions for future work.

## Background—Bell inequalities

Bell inequalities [88] were originally designed to rule out local-hidden-variable (LHV) models. They can also detect entanglement: if a bipartite state $\rho$ violates a Bell inequality, then it must be entangled. Here and in Sects. 3 and 4 we consider a simple setup, which is conventional for introducing the CHSH inequality. We call this setup the Bell-CHSH scenario. In this scenario Alice (A) and Bob (B) share a bipartite (not necessarily quantum) system, and each one of them can measure two possible observables. The observables are denoted by $A_i$ and $B_j$ for $i,j \in \left\{ 0,1 \right\}$, and their possible measurement outcomes are $\pm 1$ (hence $A_i^2 = B_j^2=1$ for all *i*, *j*). A local hidden variable $\lambda$ determines the measurement outcomes *a* and *b* via:

2 $$\begin{aligned} \Pr \left( a, b \right) = \int d\lambda \, \rho \left( \lambda \right) q \left( a \vert \lambda \right) r \left( b\vert \lambda \right) , \end{aligned}$$

where *q* and *r* are probability distributions conditioned to $\lambda$ and $\rho \left( \lambda \right)$ is a probability density function.

Let $\bar{X} :=\left( A_0, A_1, B_0, B_1 \right) ^T$ be a random vector, and let

3 $$\begin{aligned} \mathcal {B} = \mathcal {B} \left( \langle \bar{X}\rangle , \langle \bar{X}^T \bar{X} \rangle \right) \end{aligned}$$

be a (real-valued) function of the expectation values and two-point correlations of the observables. Such a function $\mathcal {B}$ is called a *Bell parameter*. Consider inequalities of the form:

4 $$\begin{aligned} \mathcal {B} \le \beta . \end{aligned}$$

If (4) holds for any LHV model for the statistics of $A_i, B_j$, then it defines a *Bell inequality*.

A quantum-mechanical model can violate a Bell inequality; the Tsirelson bound dictates the largest possible violation. Consider values $t \ge \beta$ such that $\mathcal {B} \le t$ holds for any quantum-mechanical model. We call the minimal *t* the *Tsirelson bound* of the Bell parameter $\mathcal {B}$. An inequality $\mathcal {B} \le t$ which holds for any quantum-mechanical model is also known as a *Quantum bound* [26].

Some Bell parameters are only functions of the correlations $c_{ij} :=\langle A_i B_j\rangle$. For example, the Bell-CHSH inequality:

5 $$\begin{aligned} \left| \mathcal {B}_\textrm{CHSH}\right| = \left| c_{00} + c_{01} + c_{10} - c_{11} \right| \le 2 , \end{aligned}$$

with Tsirelson bound $2 \sqrt{2}$.

Physical assumptions can constrain the values of Bell parameters. We already saw one example: assuming an LHV model, $\left| \mathcal {B}_\textrm{CHSH}\right| \le 2$. It is natural to seek other physical assumptions that may explain the values of Tsirelson bounds. Relativistic causality was suggested as such a physical principle. Let us describe its most naïve version. Alice and Bob both choose which of their possible measurements to perform; i.e., they choose *i*, *j*. If the value of *j* affects the local statistics that Alice measures, then Bob can signal a bit $j \in \left\{ 0, 1 \right\}$ to Alice arbitrarily fast, regardless of their spatial separation. Therefore, we define *no-signaling* as the condition that Alice’s marginal probability distribution is independent of *j*; and similarly, Bob’s marginal probability distribution is independent of *i*. Let $\Pr \left( A_i = a, B_j = b \mid i,j \right)$ denote the probability distribution conditioned on Alice and Bob choosing *i* and *j*, and let $\Pr \left( A_i = a \mid i,j \right) , \Pr \left( B_j = b \mid i,j \right)$ be the marginal probabilities. No-signaling means:

6 $$\begin{aligned} {\left\{ \begin{array}{ll} \Pr \left( A_i = a \mid i=0, j=0 \right) = \Pr \left( A_i = a \mid i=0, j=1 \right) \quad \forall a \\ \Pr \left( A_i = a \mid i=1, j=0 \right) = \Pr \left( A_i = a \mid i=1, j=1 \right) \quad \forall a \\ \Pr \left( B_j = b \mid i=0, j=0 \right) = \Pr \left( B_j = b \mid i=1, j=0 \right) \quad \forall b \\ \Pr \left( B_j = b \mid i=0, j=1 \right) = \Pr \left( B_j = b \mid i=1, j=1 \right) \quad \forall b. \end{array}\right. } \end{aligned}$$

Popescu-Rohrlich (PR) boxes define a no-signaling model for the probabilities $\Pr \left( A_i = a, B_j = b \mid i,j \right)$, which yields $\mathcal {B}_\textrm{CHSH} = 4$ [37]. This result indicates that the no-signaling condition alone cannot explain the Tsirelson bound $\left| \mathcal {B}_\textrm{CHSH}\right| \le 2 \sqrt{2}$. Hence, if we wish to determine the strength of quantum correlations from outside the quantum formalism, we must assume another physical principle. And indeed, combining a more refined version of relativistic causality with generalized uncertainty yields the principle of RI. As we shall see in Sect. 3, this principle *does* enable to derive the Tsirelson bound, as well as several other meaningful limits on quantum correlations.

## Relativistic independence

The RI principle, introduced by Carmi and Cohen in 2019 [35], emerges from the motivation to quantitatively derive the uniqueness of quantum correlations from basic assumptions about nature. The original no-signaling assumption, aspiring to encode relativistic causality, was given a more subtle interpretation as locality of uncertainty relations (see also [89] for an interesting, complementary approach). This means that the uncertainty relations between local observables cannot depend on choices made by remote experimenters. Thus, RI combines a subtle form of relativistic causality with a very specific notion of indeterminism, applied to multipartite systems, without assuming anything else about the underlying theory. From these minimal requirements, it attempts to derive meaningful bounds on quantum correlations. We will now gradually see the general connection between uncertainty relations and the positive-semidefiniteness property of the covariance matrix.

Let us start from the simplest local scenario: Alice measures two observables $A_0$ and $A_1$ and finds that she cannot measure both of them simultaneously with perfect precision. This experimental observation can be represented by the existence of some real number $r\ne 0$ such that the $2\times 2$ covariance matrix takes the form:

7 $$\begin{aligned} L_A = \begin{pmatrix} \Delta ^2_{A_0} & r \\ r & \Delta ^2_{A_1} \end{pmatrix} \succeq 0, \end{aligned}$$

where the $\succeq 0$ notation means that the matrix is positive-semidefinite. In our case, this implies that the product of variances $\Delta ^2_{A_0}$ and $\Delta ^2_{A_1}$ is bounded from below by $r^2$, thereby generalizing the Robertson–Schrödinger uncertainty relations.

Extending to a bipartite Bell-CHSH scenario involving Alice and Bob, the generalized covariance matrix becomes:

8 $$\begin{aligned} L_{AB} = \begin{pmatrix} L_B & C(A,B) \\ C^T(A,B) & L_A \end{pmatrix} \succeq 0, \end{aligned}$$

where *C*(*A*, *B*) represents correlations (covariances) between Alice’s and Bob’s measurements, and $L_A$, $L_B$ represent local uncertainty matrices for Alice and Bob respectively. RI requires that this inequality holds, and moreover that Alice’s uncertainty relations remain unaffected by Bob’s measurement choices and vice versa. Quantitatively:

9 $$\begin{aligned} \begin{pmatrix} \Delta ^2_{B_j} & C(B_j,A_1) & C(B_j,A_0) \\ C(B_j,A_1) & \Delta ^2_{A_1} & r_A \\ C(B_j,A_0) & r_A & \Delta ^2_{A_0} \end{pmatrix} \succeq 0, \quad \begin{pmatrix} \Delta ^2_{A_i} & C(A_i,B_1) & C(A_i,B_0) \\ C(A_i,B_1) & \Delta ^2_{B_1} & r_B \\ C(A_i,B_0) & r_B & \Delta ^2_{B_0} \end{pmatrix} \succeq 0, \end{aligned}$$

that is, $r_A$ and $r_B$ do not depend on the choices *j*, *i*, respectively. As it turns out, this assumption directly restricts quantum correlations and yields the well-known TLM bound (derived independently by Tsirelson, Landau and Masanes [38–40]):

10 $$\begin{aligned} |\varrho _{00}\varrho _{10}-\varrho _{01}\varrho _{11}| \le \sum _{j=0,1}\sqrt{(1-\varrho _{0j}^2)(1-\varrho _{1j}^2)}, \quad |\varrho _{00}\varrho _{01}-\varrho _{10}\varrho _{11}| \le \sum _{i=0,1}\sqrt{(1-\varrho _{i0}^2)(1-\varrho _{i1}^2)}, \end{aligned}$$

where $\varrho _{ij}$ are the Pearson correlations:

11 $$\begin{aligned} \varrho _{ij} {\mathop {=}\limits ^{\tiny def }}\frac{\langle A_i B_j \rangle - \langle A_i \rangle \langle B_j \rangle }{\Delta _{A_i} \Delta _{B_j}} . \end{aligned}$$

This is a significant result since $\varrho _{ij}$ are obtainable within quantum mechanics iff they satisfy the TLM inequalities [40]. Importantly, this derivation is true for any theory satisfying RI.

Now, let us derive from Eq. (9) further matrix inequalities, to be used in later sections. We start by taking Schur’s complement of the first (left) matrix there, to eliminate $\Delta _{B_j}^2$:

12 $$\begin{aligned} \begin{pmatrix} \Delta _{A_1}^2 & r_A \\ r_A & \Delta _{A_0}^2 \end{pmatrix} \succeq \frac{1}{\Delta _{B_j}^2} \begin{pmatrix} C(B_j,A_1) \\ C(B_j,A_0) \end{pmatrix} \begin{pmatrix} C(B_j,A_1)&C(B_j,A_0) \end{pmatrix}. \end{aligned}$$

Defining:

13 $$\begin{aligned} \eta _A {\mathop {=}\limits ^{\tiny def }}\frac{r_A}{\Delta _{A_0}\Delta _{A_1}} \end{aligned}$$

and assuming symmetric variances $\Delta _{A_0} = \Delta _{A_1}$ (these are the ones providing the tightest bound), this reduces to:

14 $$\begin{aligned} \begin{pmatrix} 1 & \eta _A \\ \eta _A & 1 \end{pmatrix} \succeq \begin{pmatrix} \varrho _{1j} \\ \varrho _{0j} \end{pmatrix} \begin{pmatrix} \varrho _{1j}&\varrho _{0j} \end{pmatrix} \end{aligned}$$

for $j \in \{0,1\}$. This can be proven, mutatis mutandis, with respect to Bob’s local correlations. Thus, we obtain:

15 $$\begin{aligned} \begin{pmatrix} 1 & \eta _B \\ \eta _B & 1 \end{pmatrix} \succeq \begin{pmatrix} \varrho _{i0} \\ \varrho _{i1} \end{pmatrix} \begin{pmatrix} \varrho _{i0}&\varrho _{i1} \end{pmatrix} \end{aligned}$$

for $i \in \{0,1\}$. Here $\eta _A$ and $\eta _B$ are two real numbers satisfying $\left| \eta _A\right| \le 1$, $\left| \eta _B\right| \le 1$. This result means that nonlocal correlations are bounded from above by the local ones, in the sense that the difference between the left and right hand sides is a positive-semidefinite matrix.

Quantum mechanics satisfies both relativistic causality and generalized uncertainty [35], for

16 $$\begin{aligned} \eta _A = \frac{ \frac{1}{2} \langle \{A_0,A_1\} \rangle - \langle A_0 \rangle \langle A_1 \rangle }{\Delta _{A_0} \Delta _{A_1}}, \; \; \; \eta _B = \frac{ \frac{1}{2} \langle \{B_0,B_1\} \rangle - \langle B_0 \rangle \langle B_1 \rangle }{\Delta _{B_0} \Delta _{B_1}}, \end{aligned}$$

where $\{~,~\}$ stands for the anti-commutator.

Further generalizing to a tripartite scenario involving Alice, Bob, and Charlie, the covariance matrix expands to

17 $$\begin{aligned} L_{ABC} = \begin{pmatrix} L_C & C(B,C) & C(A,C) \\ C(B,C)^T & L_B & C(A,B) \\ C(A,C)^T & C(A,B)^T & L_A \end{pmatrix} \succeq 0, \end{aligned}$$

capturing correlations among all three parties. Specifically focusing on Alice’s subsystem, conditioned on Bob’s and Charlie’s measurement choices, the uncertainty matrix reduces to

18 $$\begin{aligned} L_{ABC}^{jk} = \begin{pmatrix} \Delta ^2_{C_k} & C(C_k,B_j) & C(C_k,A_1) & C(C_k,A_0) \\ C(C_k,B_j) & \Delta ^2_{B_j} & C(B_j,A_1) & C(B_j,A_0) \\ C(C_k,A_1) & C(B_j,A_1) & \Delta ^2_{A_1} & r_{jk} \\ C(C_k,A_0) & C(B_j,A_0) & r_{jk} & \Delta ^2_{A_0} \end{pmatrix} \succeq 0. \end{aligned}$$

RI again demands invariance of Alice’s uncertainty relations under Bob’s and Charlie’s remote measurement choices, reproducing the bipartite conditions and further generalizing them.

RI has significant ramifications for quantum correlations. It explicitly excludes hypothetical stronger-than-quantum correlations such PR boxes, as these violate the RI principle by allowing one experimenter’s uncertainty relations to depend nonlocally on remote measurement choices. Additionally, RI naturally implies entanglement monogamy, restricting how entangled quantum states can be simultaneously shared among multiple parties.

Quantum mechanical correlations naturally comply with these RI-derived constraints, thus explaining quantum bounds like Tsirelson’s bound and providing deeper geometric and algebraic insights into nonlocal correlations by intrinsically linking quantum nonlocality, local uncertainty, and relativistic constraints.

## Refining bipartite bounds via relativistic independence

In this section, we show how the RI framework can be employed for refining known inequalities and deriving new ones.

### Deriving refined Tsirelson bounds on the CHSH operator

We wish to derive scalar inequalities from (14) and (15). For any column vector $\boldsymbol{u}$ of length 2, we have

19a $$\begin{aligned} & \boldsymbol{u}^T \begin{pmatrix} 1 & \eta _A \\ \eta _A & 1 \end{pmatrix} \boldsymbol{u} \ge \boldsymbol{u}^T \begin{pmatrix} \varrho _{0j} \\ \varrho _{1j} \end{pmatrix} \begin{pmatrix} \varrho _{0j}&\varrho _{1j} \end{pmatrix} \boldsymbol{u}, \end{aligned}$$

19b $$\begin{aligned} & \boldsymbol{u}^T \begin{pmatrix} 1 & \eta _B \\ \eta _B & 1 \end{pmatrix} \boldsymbol{u} \ge \boldsymbol{u}^T \begin{pmatrix} \varrho _{i0} \\ \varrho _{i1} \end{pmatrix} \begin{pmatrix} \varrho _{i0}&\varrho _{i1} \end{pmatrix} \boldsymbol{u}, \end{aligned}$$

wherein $\boldsymbol{u}^T$ denotes the transpose of $\boldsymbol{u}$. Taking $\boldsymbol{u} = \left( 1,\pm 1\right) ^{T}$ and simplifying, we obtain:

20a $$\begin{aligned} & \left| \varrho _{0j} \pm \varrho _{1j}\right| \le \sqrt{2} \sqrt{1\pm \eta _A} \end{aligned}$$

20b $$\begin{aligned} & \left| \varrho _{i0} \pm \varrho _{i1}\right| \le \sqrt{2} \sqrt{1\pm \eta _B}. \end{aligned}$$

Summing the first equation over both values of *j* yields:

21 $$\begin{aligned} \left| \varrho _{00} + \varrho _{10}\right| + \left| \varrho _{01} - \varrho _{11}\right| \le \sqrt{2} \left( \sqrt{1+ \eta _A} + \sqrt{1- \eta _A}\right) = 2\sqrt{1+\sqrt{1-\eta _A^2}} {\mathop {=}\limits ^{\tiny def }}\beta \left( \eta _A \right) . \end{aligned}$$

It is easy to verify that the maximal value of the bound is $2\sqrt{2}$, obtained for $\eta _A = 0$. The left-hand side resembles the structure of the CHSH operator from (5), and applying the triangle inequality yields:

22a $$\begin{aligned} \left| \mathcal {B}_\textrm{CHSH}\right| \le \left| \varrho _{00} + \varrho _{10}\right| + \left| \varrho _{01} - \varrho _{11}\right| \le 2\sqrt{1+\sqrt{1-\eta _A^2}} \le 2\sqrt{2}. \end{aligned}$$

In a similar manner:

22b $$\begin{aligned} \left| \mathcal {B}_\textrm{CHSH}\right| \le \left| \varrho _{00} + \varrho _{01}\right| + \left| \varrho _{10} - \varrho _{11}\right| \le 2\sqrt{1+\sqrt{1-\eta _B^2}} \le 2\sqrt{2}. \end{aligned}$$

This is what we mean by refined Tsirelson-type bounds: while $2\sqrt{2}$ is the smallest *constant* bounding $\left| \mathcal {B}_\textrm{CHSH}\right|$, here our bound is the function $\beta \left( \eta \right)$. The (constant) Tsirelson bound is the maximal value of $\beta \left( \eta \right)$, obtained for $\eta = 0$. Thus, we have derived the Tsirelson bound as a direct result of relativistic causality and generalized uncertainty. But $\eta$ may also have nonzero values, yielding smaller bounds $\beta \left( \eta \right)$. For example, $\beta \left( 1 \right) = 2$, the lowest value it can take, which is also the bound under a LHV model. Recall $\eta$ is defined as the correlation between two local observables. As such, our bound is a locally-measurable quantity that bounds the CHSH operator, which is comprised of bipartite correlations. To summarize our interpretation: when the local correlations ($\eta$) are minimal, our bound is maximal (Tsirelson bound) and when the local correlations are maximal, our bound is minimal (LHV bound). Consequently, local correlations limit the extent of bipartite correlations: the more a subsystem is locally correlated within itself, the less it can be nonlocally correlated to other subsystems.

Put differently, any theory satisfying RI obeys [35]

23 $$\begin{aligned} \left( \frac{\mathcal {B}_\textrm{CHSH}}{2\sqrt{2}} \right) ^2+\left( \frac{r_A}{\Delta _{A_0}\Delta _{A_1}}\right) ^2 \le 1, \end{aligned}$$

where in quantum mechanics $r_A=\langle \{A_0,A_1\}\rangle /2 -\langle A_0\rangle \langle A_1\rangle$. A similar inequality from Bob’s perspective holds as well. The bound in Eq. (23) was verified in a recent quantum optical experiment [90], relying on both sequential [91] and joint [92] weak measurements [93], as well as the techniques developed in [94] and [95] for realizing them.

### Stronger-than-quantum correlations and the CHSH operator

Let us observe Eq. (20a). Its right hand side is independent of $B_j$ (The operator that Bob chose to measure). We may require that the right hand side should depend on our choice to measure $B_j$, denoting this dependence by $\eta _A|_{B_j}$. From the RI principle, this sort of dependence is not possible, meaning that $\eta _A|_{B_j} = \eta _A$. Neglecting RI and using the dependence on $B_j$ gives rise to a new degree of freedom in our bounds on the refined Tsirelson inequalities. For example, Eq. (21) can now be written as

24 $$\begin{aligned} \left| \mathcal {B}_\textrm{CHSH}\right| \le \left| \varrho _{00} + \varrho _{10}\right| + \left| \varrho _{01} - \varrho _{11}\right| \le \sqrt{2} \left( \sqrt{1+ \eta _A|_{B_0}} + \sqrt{1- \eta _A|_{B_1}}\right) . \end{aligned}$$

The maximal value of this bound can in principle be 4, the stronger-than-quantum bound, and is obtained when $\eta _A|_{B_0} = - \eta _A|_{B_1} = 1$. This bound is sometimes referred to as the *algebraic* bound, as it is highest possible value consistent with the constraints $A_i, B_j = \pm 1$. The algebraic bound is achieved when naively taking $c_{00} = c_{10} =c_{01} =- c_{11} = 1$, neglecting the limitations of the underlying theory.

### Additional bounds

As was shown in [35], Eqs. (14), (15) and (16) lead to familiar and new characterizations of quantum-mechanical correlations. Examples of the latter are

25a $$\begin{aligned} & \left| \varrho _{00} \varrho _{10} - \varrho _{01} \varrho _{11}\right| \le \sum _{j=0,1} \sqrt{(1 - \varrho _{0j}^2)(1- \varrho _{1j}^2) - \left( \frac{\langle \left[ A_0, A_1 \right] \rangle }{2i \Delta _{A_0} \Delta _{A_1} } \right) ^2}, \end{aligned}$$

25b $$\begin{aligned} & \left| \varrho _{00} \varrho _{01} - \varrho _{10} \varrho _{11}\right| \le \sum _{i=0,1} \sqrt{(1 - \varrho _{i0}^2)(1- \varrho _{i1}^2) - \left( \frac{\langle \left[ B_0, B_1 \right] \rangle }{2i \Delta _{B_0} \Delta _{B_1} } \right) ^2}. \end{aligned}$$

These bounds refine the TLM inequalities (10).

One can also derive from (14), (15) and (16) further bounds on the Bell-CHSH parameter:

26a $$\begin{aligned} & \left| \mathcal {B}_\textrm{CHSH}\right| \le \min \left\{ \sqrt{2} \left( \sqrt{1 + \eta _A} + \sqrt{1 - \eta _A} \right) , \; \; 2 \sqrt{2} \sqrt{1 - \left( \frac{\langle \left[ A_0, A_1 \right] \rangle }{2i \Delta _{A_0} \Delta _{A_1} } \right) ^2} \right\} \le 2 \sqrt{2} \end{aligned}$$

26b $$\begin{aligned} & \left| \mathcal {B}_\textrm{CHSH}\right| \le \min \left\{ \sqrt{2} \left( \sqrt{1 + \eta _B} + \sqrt{1 - \eta _B} \right) , \; \; 2 \sqrt{2} \sqrt{1 - \left( \frac{\langle \left[ B_0, B_1 \right] \rangle }{2i \Delta _{B_0} \Delta _{B_1} } \right) ^2} \right\} \le 2 \sqrt{2}. \end{aligned}$$

The role of local uncertainty relations in determining the nonlocal Alice–Bob correlations is evident in all of these characterizations. Using the above definitions, one may recognize (after a straightforward calculation) the relations:

27 $$\begin{aligned} \left| \frac{\langle A_0 A_1 \rangle }{\Delta _{A_0} \Delta _{A_1} } \right| ^2 = \eta _A^2 + \left( \frac{\langle \left[ A_0, A_1 \right] \rangle }{2i \Delta _{A_0} \Delta _{A_1} } \right) ^2 \le 1 , \quad \left| \frac{\langle B_0 B_1 \rangle }{\Delta _{B_0} \Delta _{B_1} } \right| ^2 = \eta _B^2 + \left( \frac{\langle \left[ B_0, B_1 \right] \rangle }{2i \Delta _{B_0} \Delta _{B_1} } \right) ^2 \le 1 \end{aligned}$$

as the Schrödinger uncertainty relations of Alice’s $A_0$ and $A_1$, and of Bob’s $B_0$ and $B_1$, respectively. These relations utilize the real and imaginary parts of the complex correlator $\langle A_0A_1\rangle$. Discussions about nonlocal correlations often overlook this expectation value of a potentially non-Hermitian operator, but the above analysis indicates its significance in bounding nonlocality. As mentioned, it is also amenable to measurement through sequential weak measurements.

### Multiplicative Bell inequalities

The procedure outlined in the previous subsection can be generalized, yielding nonlinear Bell inequalities for multiple-input scenarios.

Before describing the setup, let us introduce another version of the matrix inequalities (14), (15):

28 $$\begin{aligned} \begin{pmatrix} 1 & r_A \\ r_A & 1 \end{pmatrix} \succeq \begin{pmatrix} c_{0j} \\ c_{1j} \end{pmatrix} \begin{pmatrix} c_{0j}&c_{1j} \end{pmatrix} , \quad \begin{pmatrix} 1 & r_B \\ r_B & 1 \end{pmatrix} \succeq \begin{pmatrix} c_{i0} \\ c_{i1} \end{pmatrix} \begin{pmatrix} c_{i0}&c_{i1} \end{pmatrix}. \end{aligned}$$

One can show that RI implies these inequalities as well. Compared with (14) and (15), the above inequalities are simpler to work with, since they only involve the one- and two-point correlators, without the standard-deviation normalization. We shall use (28) in the next section; here we require the multi-input version, which we now introduce.

The setup is as follows. Alice and Bob now have *n* possible measurements each, denoted by $A_1, \ldots , A_n, B_1, \ldots , B_n$. As before, we assume $A_i, B_j$ obtain the values $\pm 1$.

Assuming a quantum-mechanical model, we denote $c_{ij} {\mathop {=}\limits ^{\tiny def }}\langle A_i B_j\rangle$ and $r_{ij} {\mathop {=}\limits ^{\tiny def }}\langle A_i A_j\rangle$ for all *i*, *j*. Note that the $c_{ij}$ are real, while $r_{ij}^* = \langle A_j A_i\rangle = r_{ji}$. The latter implies that $r_{ij}$ are the entries of a Hermitian matrix, denoted $R_A$, which is Alice’s local covariance matrix. For any $j \in \left[ n \right]$, RI implies:

29 $$\begin{aligned} R_A \succeq \boldsymbol{c}_{j} \boldsymbol{c}_{j}^T , \quad \text {where} \quad \boldsymbol{c}_{j} :=\left( \begin{array}{c} c_{1j}\\ \vdots \\ c_{nj} \end{array}\right) . \end{aligned}$$

For any $\boldsymbol{u} \in \mathbb {R}^n$ we obtain the following inequality, valid for any quantum model predicting $c_{ij}$ and $r_{ij}$:

30 $$\begin{aligned} \boldsymbol{u}^T R_A \boldsymbol{u} \ge \boldsymbol{u}^T \boldsymbol{c}_{j} \boldsymbol{c}_{j}^T \boldsymbol{u} = \left| \boldsymbol{u} \cdot \boldsymbol{c}_{j}\right| ^2 . \end{aligned}$$

For a specific choice of a set $\left\{ \boldsymbol{u}_j \right\} _{j=1}^n$, the *n*-*input multiplicative Bell parameter* is defined by [96]:

31 $$\begin{aligned} \mathcal {B}_\textrm{Mult}^{(n)} :=\prod _{j=1}^n \left| \boldsymbol{u}_j \cdot \boldsymbol{c}_{j} \right| . \end{aligned}$$

By virtue of Eq. (30), we readily obtain the quantum bound:

32 $$\begin{aligned} \mathcal {B}_\textrm{Mult}^{(n)} \le \sqrt{ \prod _{j=1}^n \boldsymbol{u}_j^T R_A \boldsymbol{u}_j } . \end{aligned}$$

We choose $\left\{ \boldsymbol{u}_j \right\} _{j=1}^n$ to be the following (unnormalized) orthogonal basis:

33 $$\begin{aligned} \boldsymbol{u}_{j} \left( l\right) = {\left\{ \begin{array}{ll} \begin{array}{c} 1\\ -j\\ 0 \end{array} & \begin{array}{c} l\le j\\ l=j+1\\ l>j+1 \end{array} \end{array}\right. } \end{aligned}$$

For this choice, it was shown in [96] that $\prod _{j=1}^n \boldsymbol{u}_j^T R_A \boldsymbol{u}_j \le \left( n! \right) ^2$. Thus, we have the quantum bound:

34 $$\begin{aligned} \mathcal {B}_\textrm{Mult}^{(n)} \le n! , \end{aligned}$$

and we also showed that it is tight (i.e. can be saturated by a quantum model). For $n=2$, the multiplicative Bell inequality is (shifting the indices):

35 $$\begin{aligned} \mathcal {B}_\textrm{Mult}^{(2)} = \left| c_{00} - c_{10} \right| \left| c_{01} + c_{11} \right| \le 2! = 2 , \end{aligned}$$

which is a multiplicative analogue of Eq. (21). For LHV models we have the tight bound $\mathcal {B}_\textrm{Mult}^{(2)} \le 1$. The above bounds were experimentally tested in [97] by employing polarization-entangled photons.

## Multipartite bounds

Multipartite correlations are far more intricate than their bipartite counterparts. In this section, we first review basic definitions; mention several features that are unique to the multipartite scenarios; introduce known multipartite bounds; and finally, derive refined bounds via RI. For a recent comprehensive review of multipartite entanglement, see [98].

### Multipartite systems

As we mentioned in the introduction, separability and entanglement are subtler in multipartite systems than in their bipartite counterparts. Here we define several separability classes applicable for multipartite systems. Consider a Hilbert space $\mathcal {H}$ of the form (1). A density matrix $\rho$ acting on $\mathcal {H}$ is *fully separable* if it can be written as:

36 $$\begin{aligned} \rho = \sum _{j=1}^J p_j \rho _j^{\left( 1 \right) } \otimes \rho _j^{\left( 2 \right) } \otimes \ldots \otimes \rho _j^{\left( N \right) }, \end{aligned}$$

where $p_j$ defines a probability distribution and $\rho _j^{\left( i \right) }$ is a density matrix acting on $\mathcal {H}_i$.

Next, we define bi-separability of a multipartite system. Consider a partition of the indices $\left[ N \right] :=\left\{ 1, \ldots , N \right\}$ into two disjoint, nonempty subsets. This induces a bi-partition:

37 $$\begin{aligned} \underbrace{A^{(1)}\otimes A^{(2)} \otimes \ldots \otimes A^{(P)}}_{X} \otimes \underbrace{A^{(P+1)} \otimes \ldots \otimes A^{(N)}}_{Y} . \end{aligned}$$

Thus, we obtain $\mathcal {H} = \mathcal {H}_X \otimes \mathcal {H}_Y$, where $\mathcal {H}_X = \bigotimes _{n=1}^P \mathcal {H}_n$ and $\mathcal {H}_Y = \bigotimes _{n=P+1}^N \mathcal {H}_n$. We say that $\rho$ is *bi-separable* if it is separable with respect to such a bi-partition. Clearly, a fully-separable state is bi-separable (with respect to any partition). If $\rho$ is *not* bi-separable (for *any* bi-partition), we say that $\rho$ is *genuine multipartite entangled*.

Here, we already observe a unique feature of the multipartite scenario. In the bipartite setting, a state can either be separable or entangled. However, in the multipartite case, a state can be fully separable or genuine multipartite entangled, but it could also be neither. For example, consider the following three-qubit pure state $\rho = |\psi \rangle \langle \psi |$:

38 $$\begin{aligned} |\psi \rangle = \frac{1}{\sqrt{2}} |0\rangle \otimes \left( |00\rangle + |11\rangle \right) . \end{aligned}$$

Let *A*, *B*, *C* denote the parties. $|\psi \rangle$ is clearly separable with respect to the partition $A \vert BC$, hence it is not genuine multipartite entangled. However, the Schmidt decomposition of $|\psi \rangle$ with respect to the partition $AB \vert C$ is:

39 $$\begin{aligned} |\psi \rangle = \frac{1}{\sqrt{2}} \left( |00\rangle \otimes |0\rangle + |01\rangle \otimes |1\rangle \right) . \end{aligned}$$

Since both Schmidt coefficients are $\frac{1}{\sqrt{2}}$, the state is maximally entangled with respect to the partition $AB \vert C$. This shows that the very same state can be both separable and (maximally) entangled, depending on the partition. Of course, this implies that $|\psi \rangle$ is neither fully separable nor genuinely multipartite entangled.

### Known multipartite bounds

As we have seen, entanglement in multipartite settings is more intricate than in a bipartite setup. Clearly, for any *fixed* bi-partition $X \vert Y$, we can apply any result that applies for bipartite systems. However, to detect genuine multipartite entanglement, we need to somehow witness bipartite entanglement for *all* bi-partitions. Since the number of inequivalent bi-partitions is $2^{N-1}-1$, it is preferable to have a more direct approach.

Consider a multipartite Bell-CHSH scenario. With the notations of the previous subsection, suppose that the *n*th party can measure the observables $A_i^{(n)}$ for $i = 0,1$. Each $A_i^{(n)}$ has measurement outcomes $\pm 1$. Here we present two similar Bell inequalities that detect multipartite entanglement—the Seevinck–Svetlichny (SS) and Mermin–Klyshko (MK) inequalities.

We present both constructions recursively, starting with SS operators $\mathcal {S}_N^{\pm }$ [99, 100]:

40 $$\begin{aligned} \mathcal {S}_N^{\pm } = \mathcal {S}_{N-1}^{\pm } A_0^{(N)} \mp \mathcal {S}_{N-1}^{\mp } A_1^{(N)} . \end{aligned}$$

Note that we dropped the $\otimes$ notation, as customary in these types of inequalities where the order of the parties is clear. Let $'$ denote the action of replacing every $A_0^{(n)}$ by $A_{1}^{(n)}$ and vice versa. The base case for the recursion is $\mathcal {S}_2^- = - \left( \mathcal {S}_2^+ \right) ' = A_0^{(1)} A_0^{(2)} + A_0^{(1)} A_1^{(2)} + A_1^{(1)} A_0^{(2)} - A_1^{(1)} A_1^{(2)}$ (the Bell-CHSH operator). Consider a bi-partition as in Eq. (37). A hidden-variable model is *local with respect to the bi-partition*, if it has the form:

41 $$\begin{aligned} \Pr \left( a_1, \ldots , a_N \right) = \int d\lambda \rho \left( \lambda \right) \, q \left( a_1, \ldots , a_P \vert \lambda \right) r \left( a_{P+1}, \ldots , a_N \vert \lambda \right) , \end{aligned}$$

where $a_n = \pm 1$ is the measured value of party $A^{(n)}$, *q* and *r* are probability distributions conditioned to $\lambda$, and $\rho \left( \lambda \right)$ is a probability density function. The following inequality holds for any such model:

42 $$\begin{aligned} \left| \langle \mathcal {S}_N^{\pm }\rangle \right| \le 2^{N-1} . \end{aligned}$$

This inequality also holds for any quantum model, where at most $N-1$ parties are entangled. Therefore, violation of this inequality indicates genuine *N*-partite entanglement. The quantum (Tsirelson) bound:

43 $$\begin{aligned} \left| \langle \mathcal {S}_N^{\pm }\rangle \right| \le 2^{N-1} \sqrt{2}, \end{aligned}$$

is achieved by GHZ states.

The MK operators are defined via [101–103]:

44 $$\begin{aligned} \mathcal {M}_N = \frac{1}{2} \mathcal {M}_{N-1} \left( A_0^{(N)} + A_1^{(N)} \right) +\frac{1}{2} \mathcal {M}'_{N-1} \left( A_0^{(N)} - A_1^{(N)} \right) . \end{aligned}$$

The base case for the recursion is $M_1=A_0^{(1)}$. For any quantum model where at most *M* parties are entangled:

45 $$\begin{aligned} \langle \mathcal {M}_N\rangle \le 2^{ \left( M-1 \right) /2 } , \end{aligned}$$

hence a violation of this bound implies at least $M+1$-partite entanglement. The *N*-partite GHZ state saturates the upper bound for any quantum system:

46 $$\begin{aligned} \langle \mathcal {M}_N\rangle = 2^{ \left( N-1 \right) /2 } . \end{aligned}$$

### Deriving refined Tsirelson bound on the SS and MK operators

For simplicity, we continue to use the Bell-CHSH scenario in this subsection. Under a bi-partition of the form of Eqs. (37), (28) holds for the joint parties *X* and *Y*, from which the following is derived (in the same manner described in Sect. 4.1, albeit for the Pearson correlations):

47a $$\begin{aligned} & \left| \langle X_iY_k\rangle \pm \langle X_jY_k\rangle \right| \le \sqrt{ 2 \pm \langle \{X_i,X_j\}\rangle }, \end{aligned}$$

47b $$\begin{aligned} & \left| \langle X_kY_i\rangle \pm \langle X_kY_j\rangle \right| \le \sqrt{ 2 \pm \langle \{Y_i,Y_j\}\rangle }, \end{aligned}$$

which we will use to derive our inequalities in this subsection.

Starting with our refined bounds on the SS operators, consider first a bipartite system of parties $A^{(1)}$ and $A^{(2)}$. The operator $\mathcal {S}_2$ is the CHSH operator and we have in a similar manner to Eqs. (22a, 22b):

48 $$\begin{aligned} \left| \langle \mathcal {S}_2^-\rangle \right| \le 2\sqrt{1+\sqrt{1-\left( \frac{1}{2} \langle \{A^{(n)}_0,A^{(n)}_1\}\rangle \right) ^2}} \le 2\sqrt{2}, \end{aligned}$$

for $n\in \{1,2\}$. Similar bounds can be derived for $\mathcal {S}_2^+$. By the recursive definition of the Svetlichny operators Eq. (40), our bounds on $\left| \langle \mathcal {S}_{N}^{\pm }\rangle \right|$ are twice the bounds on $\left| \langle \mathcal {S}_{N-1}^{\pm }\rangle \right|$ and we have the following result for every *N*-party Svetlichny operator in the quantum regime [44]:

49 $$\begin{aligned} \left| \langle \mathcal {S}_N^{\pm }\rangle \right| \le 2^{N-1} \sqrt{1+\sqrt{1-\eta _n}} \le 2^{N-1}\sqrt{2}, \end{aligned}$$

wherein $\eta _n :=\left( \frac{1}{2} \langle \{A_0^{(n)},A_1^{(n)}\}\rangle \right) ^2$, for $n\in \{1,2,\ldots ,N\}$. Note that $0 \le \eta _n \le 1$. We have not only derived the Tsirelson bound in Eq. (43) (providing the maximal value of our bound), but, as in Eqs. (22a, 22b), a tighter Tsirelson-type bound. The minimal value of our bound, achieved for $\eta = 0$, is $2^{N-1}$, which coincides with the LHV bound given by Eq. (42). The interpretation of Eqs. (22a, 22b) applies, but here local correlations from each party bound multipartite correlations between all the parties.

For even *N*, the MK operator is, up to a normalization, equivalent to the SS operator [44], and we may use our previous construction, yielding similar bounds:

50 $$\begin{aligned} \left| \langle \mathcal {M}_N\rangle \right| \le 2^{(N-2)/2} \sqrt{1+\sqrt{1-\eta _n}} \le 2^{(N-1)/2}, \end{aligned}$$

which coincides with Eq. (46).

For odd *N*, the MK operator has $2^{N-1}$ elements, half the elements of the respective SS operator, which will require to change our construction accordingly. Thus, consider a tripartite system under the bi-partition $\underbrace{A^{(1)}}_X|\underbrace{A^{(2)}A^{(3)}}_Y$. Using Eq. (47b) combined with the triangle inequality we obtain [43]:

51 $$\begin{aligned} \begin{aligned} \left| \langle \mathcal {M}_3\rangle \right| \le \, &\,\frac{1}{2}\left( \left| \langle A^{(1)}_0A^{(2)}_0A^{(3)}_1\rangle +\langle A^{(1)}_0A^{(2)}_1A^{(3)}_0\rangle \right| + \left| \langle A^{(1)}_1A^{(2)}_0A^{(3)}_0\rangle -\langle A^{(1)}_1A^{(2)}_1A^{(3)}_1\rangle \right| \right) \\&\le \frac{1}{2}\left( \sqrt{2 + \langle \{A^{(2)}_0A^{(3)}_1,A^{(2)}_1A^{(3)}_0\}\rangle } + \sqrt{2 - \langle \{A^{(2)}_0A^{(3)}_0,A^{(2)}_1A^{(3)}_1\}\rangle }\right) . \end{aligned} \end{aligned}$$

Similar bounds comprised of different combinations of the parties can be derived by using different bi-partitions. By the recursive definition of the MK operators, our bounds on $\left| \langle \mathcal {M}_{N}\rangle \right|$ are four times the bounds on $\left| \langle \mathcal {M}_{N-2}\rangle \right|$ and we have the following result for every *N*-party MK operator in the quantum regime, where *N* is odd [44],

52 $$\begin{aligned} \left| \langle \mathcal {M}_N\rangle \right| \le 2^{(N-5)/2}\left( \sqrt{2 +\chi ^{(n,m)}_+ } + \sqrt{2 - \chi ^{(n,m)}_-}\right) \le 2^{(N-1)/2}, \end{aligned}$$

wherein $\chi ^{(n,m)}_+ :=\langle \{A^{(n)}_0A^{(m)}_1,A^{(n)}_1A^{(m)}_0\}\rangle$ and $\chi ^{(n,m)}_- :=\langle \{A^{(n)}_0A^{(m)}_0,A^{(n)}_1A^{(m)}_1\}\rangle$, for $n,m\in \{1,2,\ldots ,N\}$ such that $n \ne m$. Note that $-2 \le \chi ^{(n,m)}_{\pm } \le 2$. In contrast to our local bound on the SS operator, the bound here is comprised of bipartite correlations, since $\chi$ cannot be reduced to local correlations. The MK operators, comprised of multipartite correlations between all the parties, are thus bounded by interactions between each two parties. The maximal value of our bounds is $2^{(N-1)/2}$, which coincides with Eq. (46), and is reached for $\chi ^{(n,m)}_+ = -\chi ^{(n,m)}_- = 2$. The minimal value of our bounds is 0, and is reached for $\chi ^{(n,m)}_+ = -\chi ^{(n,m)}_- = -2$. It follows from our bound that bipartite correlations within the multipartite system come at the expense of multipartite correlations.

## Relationality of quantum correlations

The perspective-dependence of quantum correlations and entanglement, in relational quantum mechanics, and specifically, within the framework of QRFs, has been studied from various viewpoints: subsystem relativity of gravitational entropy in [82, 83], second moments and uncertainties in [85–87], subsystem relativity of entropy and thermodynamics in [81], the sum of entanglement and coherence in [73]. Among the papers mentioned above, we would like to take a deeper look at two recent papers that focus on quantum correlations in the QRF approach.

The paper [81] explores how quantum correlations and entanglement depend on the chosen internal QRF. This “relativity of subsystems” arises because different QRFs induce inequivalent Tensor Product Structures (TPS) on the physical Hilbert space. Consequently, properties like entanglement and superposition generally become QRF-dependent. The authors identify conditions for invariance. The overlap between the algebras of observables describing system S relative to frames $R_1$ and $R_2$ ($\mathcal {A}^\text {phys}_{S|R_1}\cap \mathcal {A}^\text {phys}_{S|R_2}$) consists of *S*-translation invariant operators, representing internal relations within *S* that are frame-independent. Reduced states $\rho _{S|R_i}$ are QRF-invariant ($\rho _{S|R_1}=\rho _{S|R_2}$ ) if they are *S*-translation invariant, a condition shown to be sufficient but not necessary. For pure global states, the reduced states $\rho _{S|R_1}$ and $\rho _{S|R_2}$ are unitarily related (preserving entanglement spectrum and Rényi/von Neumann entropies) if and only if the global state resides in a “TPS-invariant subalgebra” –- a subalgebra whose operators transform locally under the QRF change. For mixed states, sufficient conditions involving decompositions into such invariant pure states are provided. These results clarify when correlations between S and its complement, and correlations internal to S, appear the same or different from various QRF perspectives.

The study in [73] focuses on the relationship between entanglement and subsystem coherence under QRF transformations. The entanglement entropy is defined as $\mathcal {E}_e[|\psi \rangle ]=\mathcal {S}[\rho _A]$, where $\rho _A=\text {Tr}_B\left[ |\psi \rangle _{AB}\langle \psi |\right]$ is the reduced density matrix of the subsystem *A* and $\mathcal {S}[\rho ]=-\text {Tr}[\rho \log \rho ]$ is the von Neumann entropy. The entropy of coherence is $\mathcal {C}_e=\mathcal {S}[\rho ]-\mathcal {S}[\rho _d]$, where $\rho _d$ is the diagonal part of the state in the group element basis. The main result demonstrates that for pure bipartite states undergoing ideal QRF transformations, associated with a group *G* acting on Hilbert spaces, the sum of entanglement and subsystem coherence is invariant for two pairs of measures. Specifically, $\mathcal {C}_e^{(C)}+\mathcal {E}_e^{(C)}= \mathcal {C}_e^{(A)}+\mathcal {E}_e^{(A)}$ and $\mathcal {C}_{l^2}^{(C)}+\mathcal {E}_l^{(C)}= \mathcal {C}_{l^2}^{(A)}+\mathcal {E}_l^{(A)}$, where $\mathcal {C}_{l^2}$ is the $l^2$-norm of coherence and $\mathcal {E}_l$ is the linear entropy. Related to this, it is shown that for any choice of entanglement measure $\mathcal {E}$ and coherence measure $\mathcal {C}$, if there is a QRF where a pure bipartite state has $\mathcal {E}=0$ or $\mathcal {C}=0$, under a QRF transformation it holds that $\Delta \mathcal {C}\Delta \mathcal {E}\le 0$, where $\Delta \mathcal {C}=\mathcal {C}^{(A)}-\mathcal {C}^{(C)}$ and $\Delta \mathcal {E}=\mathcal {E}^{(A)}-\mathcal {E}^{(C)}$.

Following the *perspective-neutral* approach introduced in [75], the position and momentum correlations of individual particles, and collective behavior through the entire system’s covariance matrix, are studied in [104]. Specifically, it is shown that individual particles’ moments (variances and covariances) are perspective-dependent, satisfying certain relations. In particular, the Robertson-Schrödinger position-momentum uncertainty relation is shown to be QRF-dependent. In contrast, the determinant of the total covariance matrix remains invariant under QRF transformations. Additionally, the variance-based entanglement criteria, introduced in [105–107], also turn out to be perspective-invariant. Focusing on Gaussian states, the conditions under which the purity of a subsystem is invariant under QRF transformation are studied, which is, in general, perspective-dependent.

The structure of the current section is as follows. In Sect. 6.1, a brief introduction to the perspective-neutral approach to QRF is presented. Perspective-dependence of the position and momentum second moments is discussed in Sect. 6.2.1. Purity of individual subsystems, under QRF transformations, is studied in Sect. 6.2.2. In Sect. 6.2.3, we concentrate on uncertainties associated with individual subsystems and the entire system from different perspectives. The QRF transformation of the variance-based entanglement criteria is presented in Sect. 6.2.4.

### The perspective neutral QRF framework

Here we will present the key ideas of the perspective-neutral approach to QRF [75] that we use in [104]. The total Hilbert space, called kinematical, is assumed to be a tensor product of $\mathscr {N}$ individual objects’ Hilbert spaces, $\mathcal {H}^\text {kin}=\bigotimes _I\mathcal {H}_I$, where $I\in \mathfrak {N}$ and $\mathfrak {N}:=\{A,...,\mathscr {N}\}$, with states $|\Psi \rangle ^\text {kin}\in \mathcal {H}^\text {kin}$. The *physical* states, $|\psi \rangle ^\text {phys}$ belong to the corresponding subspace, $\mathcal {H}^\text {phys}$, satisfying a certain constraint, $\hat{C}_\text {phys}|\psi \rangle ^\text {phys}=0$. The perspective-dependent state is then obtained by a projection onto some subsystem, e.g. $I$. In the following, we focus on the continuous-variable, position-momentum case, as it appears in [75].

An arbitrary state in $\mathcal {H}^\text {kin}$, in the momentum representation is of the form

53 $$\begin{aligned} \begin{aligned} |\Psi \rangle ^{\text {kin}} =&\int \prod _{I\in \mathfrak {N}} dp_I |p_I\rangle \Psi ^\text {kin}(p_A,...,p_\mathscr {N}) = \int dP |P\rangle \Psi ^\text {kin}(P), \end{aligned} \end{aligned}$$

where we have introduced $P :=\{p_I; \; I \in \mathfrak {N}\}$ and $dP=\prod _{I\in \mathfrak {N}}dp_I$. The total Hamiltonian, for the non-relativistic case, is of the form,

54 $$\begin{aligned} \hat{H}_\text {tot}=\sum _{I\in \mathfrak {N}}\frac{\hat{p}_I^2}{2m_I}+V(\{\hat{x}\}), \end{aligned}$$

where $V(\{\hat{x}\})=V(\hat{x}_A,...,\hat{x}_\mathscr {N})$. Physical states, $|\Psi \rangle ^\text {phys}$, satisfying the constraint of translation invariance,

55 $$\begin{aligned} \hat{P}_\text {tot}|\Psi \rangle ^\text {phys}=0, \end{aligned}$$

where, $\hat{C}_\text {phys}=\hat{P}_\text {tot}=\sum _{I\in \mathfrak {N}}\hat{p}_I$, are obtained using what is known as group-averaging $|\psi \rangle ^\text {phys}=\delta (\hat{P}_\text {tot})|\psi \rangle ^\text {kin}=\int dP \delta (P_\text {tot}) |P\rangle \Psi ^\text {kin}(P)\in \mathcal {H}^\text {phys}$. In the perspective-dependent view, say *A*’s, it takes the form

56 $$\begin{aligned} \begin{aligned}&|\Psi \rangle ^{\text {phys}} = \int |p_A=-p_{\bar{A}}\rangle \prod _{I\in \mathfrak {A}} dp_I |p_I\rangle \Psi ^\text {kin}(p_A=-p_{\bar{A}},...,p_\mathscr {N}), \end{aligned} \end{aligned}$$

where $$\mathfrak {A}\equiv \mathfrak {N}\setminus \{A\}$$, and,

57 $$\begin{aligned} \begin{aligned}p_{\bar{A}}=\sum _{I\in \mathfrak {A}}p_I. \end{aligned}\end{aligned}$$

Denoting $\psi _{\bar{A}}(P_{\bar{A}})= \Psi ^\text {kin}(p_A=-p_{\bar{A}},...,p_\mathscr {N})$, $dP_{\bar{A}}=\prod _{I\in \mathfrak {A}} dp_I$, and $|P_{\bar{A}}\rangle =\prod _{I\in \mathfrak {A}}|p_I\rangle$, we may write,

58 $$\begin{aligned} \begin{aligned} |\Psi \rangle ^{\text {phys}}&= \int dP_{\bar{A}}|p_A=-p_{\bar{A}}\rangle |P_{\bar{A}}\rangle \psi _{\bar{A}}. \end{aligned} \end{aligned}$$

From the perspective of a certain particle, say *I*’s, according to [75], the state is as follows,

59 $$\begin{aligned} |\psi _{\bar{I}}\rangle :=\sqrt{2\pi } \langle x_I=0|\Psi \rangle ^{\text {phys}}= \int dP_{\bar{I} }|P_{\bar{I}}\rangle \psi _{\bar{I}}, \end{aligned}$$

and the perspective-dependent Hamiltonian of the form,

60 $$\begin{aligned} \hat{H}_{\bar{A}}=\frac{\hat{p}_{\bar{A}}^2}{2m_A} + \sum _{I\in \mathfrak {A}}\frac{\hat{p}_I^2}{2m_I}+V_{\bar{A}}, \end{aligned}$$

where $V_{\bar{A}}=\Big [V(\{x\})\Big ]_{x_A=0}$, governs the dynamics of $|\psi _{\bar{A}}\rangle$, i.e.,

61 $$\begin{aligned} i\frac{d}{dt}|\psi _{\bar{A}}(t)\rangle = \hat{H}_{\bar{A}} |\psi _{\bar{A}}(t)\rangle , \end{aligned}$$

where $|\psi _{\bar{A}}(0)\rangle = |\psi _{\bar{A}}\rangle$, and, $|\psi _{\bar{A}}(t)\rangle = \int dP_{\bar{I}} e^{-itH_{\bar{A}}} |P_{\bar{A}}\rangle \psi _{\bar{A}}$. In the limit when $m_A\gg m_{I\in \mathfrak {N}{\setminus }\{A\}}$, the non-relational description is recovered, as discussed in [75].

Expectation value and variance of an operator $\hat{O}$, in the reference frame of particle *I*, are of the form,

62 $$\begin{aligned} \begin{aligned} \langle \hat{O}\rangle _I(t)=&\langle \psi _{\bar{I}}(t)|\hat{O}|\psi _{\bar{I}}(t)\rangle = \langle \psi _{\bar{I}}(t=0) e^{it\hat{H}_{\bar{I}}} |\hat{O}| e^{-it\hat{H}_{\bar{I}}} \psi _{\bar{I}}(t=0)\rangle , \end{aligned} \end{aligned}$$

and

63 $$\begin{aligned} \sigma ^2_{I}(\hat{O})= \langle \hat{O}^2\rangle _I - \langle \hat{O}\rangle _I^2. \end{aligned}$$

For the perspective-dependent covariance of two operators we use the following symmetric form

64 $$\begin{aligned} \begin{aligned}&\text {cov}_{I}(\hat{O}_1,\hat{O}_2)= \text {cov}_{I}(\hat{O}_2,\hat{O}_1)= \frac{1}{2}\left( \langle \hat{O}_1\hat{O}_2\rangle _I+\langle \hat{O}_2\hat{O}_1\rangle _I\right) - \langle \hat{O}_1\rangle _I\langle \hat{O}_2\rangle _I. \end{aligned} \end{aligned}$$

Using the above, the covariance matrix associated with $\hat{O}_1$ and $\hat{O}_2$, from *I*’s perspective, is defined as follows

65 $$\begin{aligned} \begin{aligned} \boldsymbol{\Sigma }_{(I)}^{(\hat{O}_1,\hat{O}_2)}= \left[ \begin{matrix} \sigma _I^2(\hat{O}_1)& \text {cov}_I(\hat{O}_1,\hat{O}_2)\\ \text {cov}_I(\hat{O}_2,\hat{O}_1)& \sigma _I^2(\hat{O}_2) \end{matrix}\right] . \end{aligned} \end{aligned}$$

#### Perspective-dependence of entanglement

To demonstrate the perspective-dependence of entanglement (nonlocal correlation), following the framework in the previous section, let us focus, for simplicity, on the case of three subsystems, *A*, *B* and *C*, described from the perspective of the fourth, *D*. Such a state, in momentum representation, is of the form:

66 $$\begin{aligned} |\psi _{\bar{D}}\rangle =\int dp_Adp_Bdp_C\Psi ^\text {kin}(p_A,p_B,p_C,-p_A-p_B-p_C)|p_A,p_B,p_C\rangle . \end{aligned}$$

**Fig. 1:**
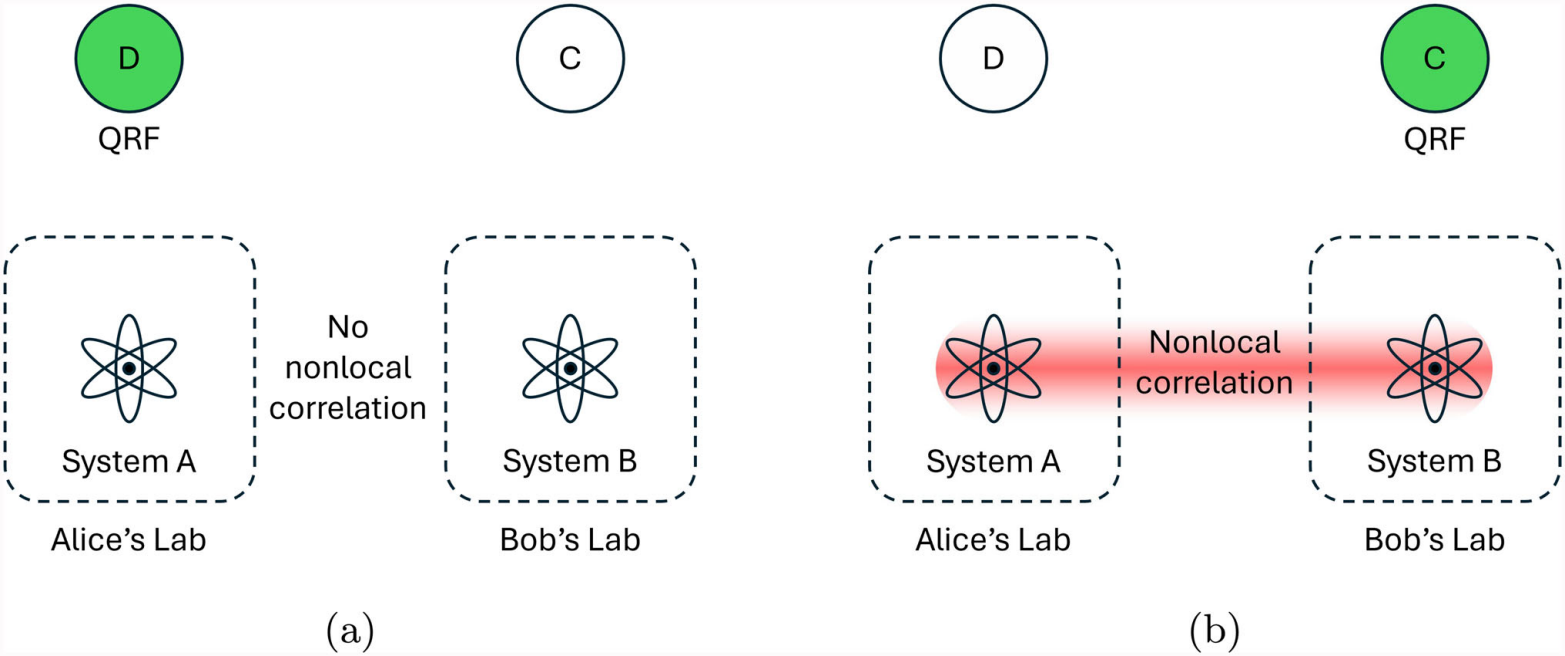
Illustration of the QRF-dependence of entanglement (non-locality). In (a), systems *A* and *B* are in a separable state from *D*’s perspective, when, from *C*’s perspective, in (b), they are entangled

As was shown in [70, 75], if the subsystems in *D*’s QRF are found in a separable state, illustrated in Fig 1a

67 $$\begin{aligned} |\psi _{\bar{D}}\rangle =\int dp_Adp_Bdp_C\phi (p_A)\eta (p_B)\xi (p_C)|p_A,p_B,p_C\rangle , \end{aligned}$$

the ones in *C*’s QRF, namely, *A*, *B* and *D*, are entangled, as illustrated in Fig. 1b:

68 $$\begin{aligned} |\psi _{\bar{C}}\rangle =\int dp_Adp_Bdp_D \phi (p_A)\eta (p_B)\xi (-p_A-p_B-p_D)|p_A,p_B,p_D\rangle . \end{aligned}$$

### Initial instant invariants and frame-dependent quantities

#### Position and momentum second moments

The reciprocal position variances of any two QRFs coincide,

69 $$\begin{aligned} \begin{aligned} \sigma ^2_I\left( \hat{x}_J\right) = \sigma ^2_J\left( \hat{x}_I\right) , \end{aligned}\end{aligned}$$

when their descriptions of other particles do not [104],

70 $$\begin{aligned} & \sigma _I^2(\hat{x}_K)-\sigma _J^2(\hat{x}_K)= \text {cov}_I(\hat{x}_J,\hat{x}_K)-\text {cov}_J(\hat{x}_I,\hat{x}_K), \end{aligned}$$

71 $$\begin{aligned} & \text {cov}_I(\hat{x}_K,\hat{x}_L)- \text {cov}_J(\hat{x}_K,\hat{x}_L)= \frac{1}{2}\left[ \left( \sigma _I^2(\hat{x}_K)-\sigma _J^2(\hat{x}_K)\right) + \left( \sigma _I^2(\hat{x}_L)-\sigma _J^2(\hat{x}_L)\right) \right] . \end{aligned}$$

where $I,J,K,L\in \mathfrak {N}$. For any three particles, the relations between the position second moments are equivalent to those in a Euclidean triangle. In this analogy, the variances, $\sigma _a(\hat{x}_b)$, represent the side lengths, and the opposite angles, $\alpha _c(\hat{x}_a,\hat{x}_b)$, are related to the correlations, $\cos (\alpha _a(\hat{x}_b,\hat{x}_c))=\text {corr}_a(\hat{x}_b,\hat{x}_c)$, satisfying cosine laws

72 $$\begin{aligned} \begin{aligned}&\sigma ^2_a(\hat{x}_b)= \sigma ^2_c(\hat{x}_a)+\sigma ^2_c(\hat{x}_b)- 2\sigma _c(\hat{x}_a)\sigma _c(\hat{x}_b) \cos (\alpha _a(\hat{x}_b,\hat{x}_c)), \end{aligned} \end{aligned}$$

and the following inequalities regarding the variances,

73 $$\begin{aligned} \begin{aligned} |\sigma _c(\hat{x}_a)-\sigma _c(\hat{x}_b)| \le \sigma _a(\hat{x}_b) \le \sigma _c(\hat{x}_a)+\sigma _c(\hat{x}_b), \end{aligned}\end{aligned}$$

and correlations

74 $$\begin{aligned} \text {corr}_a(\hat{x}_b,\hat{x}_c) \text {corr}_b(\hat{x}_c,\hat{x}_a)+ \text {corr}_c(\hat{x}_a,\hat{x}_b)\ge 0, \end{aligned}$$

where $$a\ne b\ne c\in \left\{ I,J,K\right\}$$, and

75 $$\begin{aligned} \text {corr}_a(\hat{x}_b,\hat{x}_c)+\text {corr}_d(\hat{x}_e,\hat{x}_f)\ge 0, \end{aligned}$$

where $d\ne e\ne f\in \left\{ I,J,K\right\}$. In addition, we have bounds on the correlations’ sum

76 $$\begin{aligned} 1\le \text {corr}_I(\hat{x}_J,\hat{x}_K)+ \text {corr}_J(\hat{x}_K,\hat{x}_I)+ \text {corr}_K(\hat{x}_I,\hat{x}_J)\le 3/2, \end{aligned}$$

and product

77 $$\begin{aligned} -1\le \text {corr}_I(\hat{x}_J,\hat{x}_K) \text {corr}_J(\hat{x}_K,\hat{x}_I) \text {corr}_K(\hat{x}_I,\hat{x}_J)\le 1/8. \end{aligned}$$

Regarding the momentum second moments, the descriptions of other particles coincide in different QRFs:

78 $$\begin{aligned} \begin{aligned} \sigma ^2_I(\hat{p}_K)=\sigma ^2_J(\hat{p}_K), \end{aligned} \end{aligned}$$

and,

79 $$\begin{aligned} \text {cov}_{I}(\hat{p}_K,\hat{p}_L)= \text {cov}_{J}(\hat{p}_K,\hat{p}_L), \end{aligned}$$

when the reciprocal ones, when the new QRF is the one that is described in the previous one, do not, and are related as follows:

80 $$\begin{aligned} \begin{aligned} \sigma ^2_{a\ne I}(\hat{p}_I)=&\sigma ^2_{I}(\hat{p}_{\bar{I}})= \sum _{b,c \ne I}\text {cov}_I(\hat{p}_b,\hat{p}_c)= \sum _{b\ne I}\sigma ^2_I(\hat{p}_b)+ \sum _{b\ne c \ne I}\text {cov}_I(\hat{p}_b,\hat{p}_c), \end{aligned} \end{aligned}$$

and

81 $$\begin{aligned} \begin{aligned}&\text {cov}_{a\ne I,J}(\hat{p}_I,\hat{p}_J)= -\text {cov}_{b}(\hat{p}_{\bar{b}},\hat{p}_c)= -\sigma _b^2(\hat{p}_c) -\sum _{d\ne I,J}\text {cov}_b(\hat{p}_d,\hat{p}_c)= -\sigma _b^2(\hat{p}_c)- \text {cov}_b(\hat{p}_{\overline{IJ}},\hat{p}_c), \end{aligned} \end{aligned}$$

where $b,c=I,J$, and $\hat{p}_{\overline{IJ}}=\sum _{K\ne I,J}\hat{p}_K$ is the momentum of the rest of the system, excluding particles *I* and *J*. Using Eq. (81) we obtain the following inequalities of the momentum second moments:

82 $$\begin{aligned} \begin{aligned} |\sigma _I(\hat{p}_J)-\sigma _J(\hat{p}_I)|\le \sigma _{I,J}(\hat{p}_{\overline{IJ}}), \end{aligned} \end{aligned}$$

and,

83 $$\begin{aligned} \begin{aligned}&\text {corr}_a(\hat{p}_{ \overline{IJ}},\hat{p}_b) \le \text {corr}_b(\hat{p}_{\overline{IJ}},\hat{p}_a) \text {cov}_{c\ne I,J}(\hat{p}_a,\hat{p}_b). \end{aligned}\end{aligned}$$

#### Purity and equivalent QRFs

In the current study, the total state of the system is assumed to be pure from any perspective. In general, subsystems of a multipartite system, described by a pure state, are represented by mixed states. In the following, we address the question of whether different QRFs agree on the purity of the same subsystem from their perspectives, limiting the discussion to the case of Gaussian states. In this case, the purity is determined by the determinant of the covariance matrix:

84 $$\begin{aligned} \mu (\rho )=\frac{1}{2^n\sqrt{\det {\boldsymbol{\Sigma }}}}. \end{aligned}$$

The determinants of the position-momentum covariance matrices, associated with a certain particle, say *K*, from different perspectives, say *I*’s and *J*’s, do not coincide, in general,

85 $$\begin{aligned} \begin{aligned}&\det {\boldsymbol{\Sigma }}_{(I)\left\{ K\right\} }^{(x,p)}= \sigma _I^2(\hat{x}_K)\sigma _I^2(\hat{p}_K)- \text {cov}_I(\hat{x}_K,\hat{p}_K) \text {cov}_I(\hat{p}_K,\hat{x}_K)=\left[ \sigma _J^2(\hat{x}_K)+ \sigma _J^2(\hat{x}_I)-2\text {cov}_J(\hat{x}_I,\hat{x}_K) \right] \sigma _J^2(\hat{p}_K)\\&\quad -\left[ \text {cov}_J(\hat{x}_K,\hat{p}_K)- \text {cov}_J(\hat{x}_I,\hat{p}_K) \right] \left[ \text {cov}_J(\hat{p}_K,\hat{x}_K)-\text {cov}_J(\hat{p}_K,\hat{x}_I) \right] \ne \det {\boldsymbol{\Sigma }}_{(J)\left\{ K\right\} }^{(x,p)}. \end{aligned} \end{aligned}$$

Hence, in general, different QRFs do not agree on the purity of the same subsystem, due to correlations of the QRFs with the described object, from each others’ perspectives, $\mathrm{cov}_J(\hat{x}_I,\hat{x}_K)$, $\text {cov}_J(\hat{x}_I,\hat{p}_K)$, and reciprocal position variance, $\sigma _J^2(\hat{x}_I)$.

According to Eqs. (69)–(71) one can see that if *I* and *J* are approximately localized in each others’ frames, i.e. $\sigma _I^2(\hat{x}_J)\approx 0$, then the spatial moments of all the other particles coincide from their perspectives:

86 $$\begin{aligned} & \sigma _I^2(\hat{x}_K)\approx \sigma _J^2(\hat{x}_K), \end{aligned}$$

87 $$\begin{aligned} & \text {cov}_I(\hat{x}_K,\hat{x}_L)\approx \text {cov}_J(\hat{x}_K,\hat{x}_L), \end{aligned}$$

and, their covariances with other particles, from each other’s perspectives, vanish,

88 $$\begin{aligned} \begin{aligned} \text {cov}_I(\hat{x}_J,\hat{x}_K)\approx \text {cov}_J(\hat{x}_I,\hat{x}_K) \approx 0, \end{aligned}\end{aligned}$$

where $K,L\in \mathfrak {N}\setminus \left\{ I,J\right\}$. Additionally, if the mixed position-momentum covariances vanish in both QRFs,

89 $$\begin{aligned} \text {cov}_J(\hat{x}_I,\hat{p}_K)\approx \text {cov}_I(\hat{x}_J,\hat{p}_K)\approx 0, \end{aligned}$$

that gives

90 $$\begin{aligned} \text {cov}_J(\hat{x}_K,\hat{p}_L)\approx \text {cov}_I(\hat{x}_K,\hat{p}_L). \end{aligned}$$

Since, in this case, according to Eqs. (78), (79), (86), (87) and (90) QRFs *I* and *J* agree on all the position, momentum and mixed second moments of the described particles, they may be considered equivalent in describing any subsystem within a Gaussian state. Therefore, they are also equivalent when addressing the corresponding purity.

#### Uncertainties

According to Eqs. (70) and (78), the position-momentum uncertainty relation does not coincide in different QRFs:

91 $$\begin{aligned} \begin{aligned} \sigma _I^2(\hat{x}_K)\sigma _I^2(\hat{p}_K)\ne \sigma _J^2(\hat{x}_K)\sigma _J^2(\hat{p}_K). \end{aligned}\end{aligned}$$

However, as was shown in [104], the determinants of the total position and momentum covariance matrices are invariant under switching perspectives,

92 $$\begin{aligned} \det \widetilde{\boldsymbol{\Sigma }}^{(r)}_{(A)}= \det \widetilde{\boldsymbol{\Sigma }}^{(r)}_{(B)}, \end{aligned}$$

where $r=x,p$, as well as the of combined position-momentum one,

93 $$\begin{aligned} \det \boldsymbol{\Sigma }^{(x,p)}_{(A)}= \det \boldsymbol{\Sigma }^{(x,p)}_{(B)}. \end{aligned}$$

That means that the uncertainty volume, associated with the determinant of the total covariance matrix, is invariant under changing perspectives.

#### Variance-based entanglement criteria

The well-known variance-based entanglement criteria utilize EPR-like operators, $\hat{x}_1-\hat{x}_2$ and $\hat{p}_1+\hat{p}_2$. By analyzing the variances of these operators, one can derive criteria based on a product, introduced in [105] and a sum, introduced in [106, 107]. For Gaussian states, which are entirely described by their second moments, separability is assured if

94 $$\begin{aligned} \text {C}^\text {prod}_{12}:=\sigma ^2(\hat{x}_1-\hat{x}_2)\sigma ^2(\hat{p}_1+\hat{p}_2)\ge 1, \end{aligned}$$

or,

95 $$\begin{aligned} \text {C}^\text {sum}_{12}:=\sigma ^2(\hat{x}_1-\hat{x}_2)+\sigma ^2(\hat{p}_1+\hat{p}_2)\ge 2. \end{aligned}$$

In other words, any violation of these inequalities is a definitive indication of entanglement. For non-Gaussian states, however, satisfying the inequalities does not guarantee separability because important information may reside in higher-order moments. Nonetheless, their violation still provides a sufficient condition for the presence of entanglement [105–107].

In the spatial QRF formalism, described here, the momentum moments coincide in all QRFs; hence

96 $$\begin{aligned} \sigma _I^2(\hat{p_K}+\hat{p}_L)= \sigma _J^2(\hat{p_K}+\hat{p}_L). \end{aligned}$$

Additionally, the variance of the positions’ difference of any two particles is perspective-invariant,

97 $$\begin{aligned} \sigma _I^2(\hat{x_K}-\hat{x}_L)= \sigma _J^2(\hat{x_K}-\hat{x}_L), \end{aligned}$$

Hence, both the product (94) and the sum (95) criteria are invariant under QRF transformations, at the initial instant,

98 $$\begin{aligned} \text {C}^\text {prod/sum}_{(I)KL}= \text {C}^\text {prod/sum}_{(J)KL}. \end{aligned}$$

This means that if any two particles are entangled according to the variance-based conditions from one perspective, it is true from any other.

## Conclusion

In this review article we revisited several aspects of quantum correlations, presenting various bounds on bipartite and multi-partite correlations, as well as the ways to derive them. Special emphasis was given to relational aspects and to QRFs. We have seen that despite violating the assumption of local-realism, quantum theory obeys a specific type of locality constraint pertaining to uncertainty relations. This condition, known as Relativistic Independence, ensures relativistic causality at the level of generalized uncertainty relations. As a consequence, several new bounds on quantum correlations were derived, all having a fundamental property in common—they show how local and nonlocal correlations mutually bound each other. Interestingly, several of these bounds were tested in recent quantum optical experiments [90, 97], providing empirical characterizations of the quantum correlations set.

Uncertainties and quantum correlations were shown to be frame-dependent, in general, satisfying specific relations and inequalities between different perspectives. However, the fundamental notion underlying this review article, namely the determinant of the total covariance matrix, was proven to be invariant under changes of QRF (as opposed to those associated with individual subsystems). Similarly, variance-based entanglement criteria were shown to be perspective-independent.

The QRF formalism—which promotes the frames of reference, with respect to which observations are made, to quantum objects—provides an additional layer of relational intricacy to the principle of Relativistic Independence discussed in the first part of this review. Similarly to the original RI framework, QRFs are focused on what changes and what remains invariant under change of perspective, but the set of changes they allow is much larger. We also see the significance of the covariance matrix in both cases and utilize similar mathematical techniques based on positive-semidefiniteness and the ensuing bounds on second moments.

Finally, alongside this intensively studied type of “kinematic” (i.e. Bell-like) nonlocality, we wish to draw attention to the less explored type of dynamical nonlocality [108–111], related to the time evolution of states and operators which bears unique quantum features, i.e. nonlocal equations of motion. This type of nonlocality seems to propose additional interesting avenues to examine, especially in the context of QRFs [112].

## Data Availability

Not applicable.
